# Developing flexible models for genetic evaluations in smallholder crossbred dairy farms

**DOI:** 10.3168/jds.2022-23135

**Published:** 2023-12

**Authors:** R. Costilla, J. Zeng, M. Al Kalaldeh, M. Swaminathan, J.P. Gibson, V. Ducrocq, B.J. Hayes

**Affiliations:** 1 AgResearch Limited, Ruakura Research Centre, Hamilton 3214, New Zealand; 2 Centre for Animal Science, Queensland Alliance for Agriculture and Food Innovation, University of Queensland, St. Lucia, QLD 4067, Australia; 3 Institute for Molecular Biosciences, University of Queensland, St. Lucia, QLD 4067, Australia; 4 Centre for Genetic Analysis and Applications, School of Environmental and Rural Science, University of New England, Armidale, NSW 2350, Australia; 5 BAIF Development Research Foundation, Pune 412 202, Maharashtra, India; 6 Universite Paris-Saclay, INRAE, AgroParisTech, UMR GABI, 78350 Jouy-en-Josas, France

**Keywords:** milk yield, developing countries, BayesR, QTL, GWAS

## Abstract

The productivity of smallholder dairy farms is very low in developing countries. Important genetic gains could be realized using genomic selection, but genetic evaluations need to be tailored for lack of pedigree information and very small farm sizes. To accommodate this situation, we propose a flexible Bayesian model for the genetic evaluation of milk yield, which allows us to simultaneously account for nongenetic random effects for farms and varying SNP variance (BayesR model). First, we used simulations based on real genotype data from Indian crossbred dairy cattle to demonstrate that the proposed model can separate the true genetic and nongenetic parameters even for small farm sizes (2 cows on average) although with high standard errors in scenarios with low heritability. The accuracy of genomic genetic evaluation increased until farm size was approximately 5. We then applied the model to real data from 4,655 crossbred cows with 106,109 monthly test day milk records and 689,750 autosomal SNPs. We estimated a heritability of 0.16 (0.04) for milk yield and using cross-validation, a genomic estimated breeding value (GEBV) accuracy of 0.45 and bias (regression of phenotype on GEBV) of 1.04 (0.26). Estimated genetic parameters were very similar using BayesR, BayesC, and genomic BLUP approaches. Candidate genes near the top variants, *IMMP2L* and *ARHGEF2*, have been previously associated with milk protein composition, mastitis resistance, and milk cholesterol content. The estimated heritability and GEBV accuracy for milk yield are much lower than those from intensive or pasture-based systems in many countries. Further increases in the number of phenotyped and genotyped animals in farms with at least 2 cows (preferably 3–5, to allow for dropout of cows) are needed to improve the estimation of genetic effects in these smallholder dairy farms.

## INTRODUCTION

The productivity of smallholder dairy farms is very low in developing countries for several reasons, including genetics, environmental conditions, management, and government regulations. For instance, in India, despite being the world's largest milk producer, the milk yield per cow is only one-eighth of the levels achieved in the United States and Canada (OECD and FAO, 2019) with predominantly low-input/low-yield dairy production systems (Hemme and Deeken, 2007; Morgan, 2009).

In principle, substantial genetic gains could be realized using new breeding technologies such as genomic selection, especially in smallholder systems (Burrow et al., 2021). Recent experiences for crossbred dairy cattle in Africa (Brown et al., 2016; Ojango et al., 2019; Mrode et al., 2021) and India (Al Kalaldeh et al., 2021) show that genetic progress is possible with genomic selection even in these challenging conditions.

Appropriate modeling for genetic evaluations in smallholder systems is also challenging, as these systems typically have no pedigree information, weak genetic connectedness, and very small farm sizes. An important question is, then, how well can we separate genetic from management effects in smallholder farms? Current efforts that use genomic BLUP (**GBLUP**) approaches in these contexts (Ojango et al., 2019; Al Kalaldeh et al., 2021; Mrode et al., 2021) might not be the most accurate approaches if there are some QTL of moderate to large effect (e.g., *DGAT1* for fat percentage; Grisart et al., 2004). For instance, the BayesR model has provided more accurate predictions for several traits for dairy and tropically adapted beef cattle (Kemper et al., 2015; Hayes et al., 2019).

The question of whether such models can disentangle genetic effects from farm effects when farm sizes are very small has been investigated using simulation (Powell et al., 2021). The authors found that modeling farm effects as random had higher accuracies than modeling them as fixed at small farm sizes (≤4 cows). However, the simulation approach of Powell et al. (2021) assumes known genetic parameters and can only approximate admixture levels in real crossbred populations.

Here we propose a flexible Bayesian model that allows us to simultaneously account for nongenetic random effects for farms and varying SNP variance, a modest extension of the previously described BayesR model (Erbe et al., 2012; Kemper et al., 2015; Moser et al., 2015). Additionally, BayesR directly produces a prediction equation that can be applied to any new animal as soon as it is genotyped, speeding up selection decisions from DNA sampling to breeding values.

To answer our research question, “How well can we separate genetic from management effects in smallholder farms?” we used simulations based on real genotype data from crossbred dairy cows from India. We were particularly interested to examine whether the proposed model can separate the true genetic and nongenetic effects despite the small sample sizes in these contexts. Finally, we applied the BayesR model to existing monthly test day milk records from Indian cows, compared it with the GBLUP and BayesC (Habier et al., 2011) approaches, and used cross-validation to calculate the accuracy and bias for the genomic EBV (**GEBV**).

## MATERIALS AND METHODS

This study was based on existing data and no live animals were used. Animal ethical approval was therefore not required.

### Phenotypic and Genotypic Records

The phenotypic and genotypic data were previously collected by the BAIF Development Research Foundation (https://baif.org.in), a nongovernmental organization established in 1967 with a mission to enhance livelihoods of Indian rural families, using smallholder dairy production as one of the tools to achieve this objective. We used monthly test day (**TD**) milk records (L/d) collected from Indian smallholder dairy farms. Existing records from 4,655 cows with 106,109 monthly TD milk records and 689,750 autosomal imputed SNPs (Al Kalaldeh et al., 2021) were used here for our analyses. Cows were crossbreds between local indigenous *Bos indicus* cattle and exotic dairy breeds, mainly Holstein/Friesian and Jersey (Strucken et al., 2021). The average breed composition of these animals was estimated to be 0.48 Holstein/Friesian, 0.15 Jersey, and 0.37 indigenous (Al Kalaldeh et al., 2021). Milk yield records came from 6 Indian states (Bihar, Jharkhand, Maharashtra, Odisha, Punjab, and Uttar Pradesh) between 2016 and 2020.

The TD records were collected from crossbred cows raised in smallholder farms, ranging in size from 1 to 43 cows, with an average size of 1.7 animals per farm. All TD records were corrected for fixed effects, including cattle development center (**CDC**), season, the interaction of CDC by season, parity, lactation curves for parities with a third-order Legendre polynomial, and lactation curves for CDC modeled with a third-order Legendre polynomial. Fixed effects for breed were obtained as the regressions on breed proportion. An estimate of the production environment that each cow experiences was obtained as the sum of the estimated fixed CDC effect and random farm effect of that cow. The environmental estimates were then ordered and classified into bottom, middle, and top thirds, creating a fixed effect of production environment with 3 levels: low, medium, and high environments. Further details about trait collection, construction of corrected phenotypes, breed percentages, and production environments can be found in Al Kalaldeh et al. (2021). Adjusted TD records are averaged by cow and used as a phenotype for the all genetic models.

### Simulations

We performed simulations using real genotype data from BAIF from smallholder farms in India. For the genotypes, we randomly chose 50,000 genome-wide markers from the imputed HD genotypes for all available 4,655 crossbred cows (Al Kalaldeh et al., 2021). The phenotypes were generated using a model with 5,000 causal markers with varying SNP variance, farm sizes, and heritabilities. The QTL effects for the causal markers were sampled from a mixture of normal distributions:

0.80 × *N*(0,10^−4^) + 0.15 × *N*(0,10^−3^) + 0.04 × *N*(0,10^−2^) + 0.01 × (0,10^−1^).

The simulated phenotypes were generated using GCTA v.1.94.1 (Yang et al., 2011).

The mixture distribution for the SNP effects allows the estimation of QTL of varying effects; that is, few markers with large effects and many markers with very small effect. Farm sizes were sampled from a truncated Poisson distribution with means equal to 1, 2, 5, and 20 (average farm size). Farm effects followed a normal distribution with zero mean and a farm variance equal to 3 times the additive genetic variance. We set $\sigma_{f}^{2}=3\sigma_{g}^{2}$ based on the estimates obtained by Al Kalaldeh et al. (2021) and other studies in smallholder contexts where the farm or environmental variance is much larger than the additive genetic variance. We used 3 values for the heritabilities (*h*^2^ = 10%, 20%, and 50%) for a total of 12 scenarios. For each scenario, we generated 50 simulated datasets, giving a total of 600 simulated datasets. Breed effects were not incorporated in the simulations.

Model estimation for the simulated data used Markov chain Monte Carlo (**MCMC**) chains of 5,000 iterations, 2,000 burn-in, and 5 thinning. For each scenario, we present the estimated genetic parameters, additive genetic variance, and heritabilities. Models also included weights for the residuals based on the real number of phenotypic records per animal (Equation 2).

### Validation for the Simulations

In addition to the estimation of genetic parameters, GEBV from simulations were validated using prediction accuracies and bias from cross-validation (5-fold). Prediction accuracy was calculated as the Pearson linear correlation (r) between the simulated phenotype and GEBV, and bias as the regression of the simulated phenotype on the GEBV.

### Real Data Application

For the real data application, we fitted a BayesR model with random farm effects (model 1, Equation 1), plus another model that added production environment (low, medium, high) as fixed effect (model 2). Model parameters for the real data application were estimated using 3 MCMC chains each with 25,000 iterations, 5,000 burn-in, and 5 thinning. This means that (25,000 − 5,000)/5 = 4,000 iterations per MCMC chain were used for inference. The MCMC convergence was assessed using the multivariate version of the Gelman-Rubin diagnostic (Gelman and Rubin, 1992). Chains were combined and visualized using the R package coda (Plummer et al., 2006).

Functional annotation of markers with the highest posterior inclusion probability (**PIP**) in the BayesR model was carried using the Variant Effect Predictor tool from Ensembl, release 107 (Ensembl, 2022). We annotated the top 10 variants with the highest PIP. Candidate genes were also mapped in Ensembl using a 100-kb window from markers.

### Validation for the Real Data Application

Validation for the real data application was carried out using cross-validation of multi-breed groups. The validation was performed across CDC, which are made up of geographically close villages within Indian districts and states, with all animals within a CDC within either the reference set or the validation set. This validation strategy was a 10-fold random cross-validation, where all 87 CDC across India were partitioned in 10 random groups (9 groups of 9 CDC and 1 group of 6 CDC). In turn, each group of CDC was then taken as a validation set, the remaining as the reference set. The SNP effects were calculated from the reference set, and GEBV were calculated. Prediction accuracy was calculated as the Pearson linear correlation (r) of the GEBV and the phenotype adjusted for fixed effects. Bias was calculated as the regression of GEBV on adjusted phenotype. To compare with selection based on phenotypes only, we calculated the prediction accuracy of mass selection for repeated records using the equation rh2/rh2[1+(r−1)t][1+(r−1)t], where *r* is the number of records per animal, and *h*^2^ and *t* are the trait heritability and repeatability, respectively (Mrode, 2014). We used *r* = 18 (average number of records per animal in the Indian real data application), *t* = 0.60, and *h*^2^ = 0.19 based on previous estimates for India (Table 3 in Al Kalaldeh et al., 2021).

### Estimation Model

For both the simulations and real data application, we used the same genetic model, a BayesR model (Erbe et al., 2012) with random farm effects:

[1] $$y=1_{n}m+Vf+Zg+e,$$

where **y** is a vector of *n* adjusted phenotypes, **1_n_** is a vector of ones, *m* is an overall mean, **f** is a vector of *p* farm effects with distribution $f\&sim;N\left(0,\sigma_{f}^{2}\right),$ and **g** is a vector of *m* SNP effects with distribution $g\&sim;N\left(0,\sigma_{i}^{2}\right),$ where *i* = 1, . . . ,4. The variance for each SNP was assumed to be from 1 of 4 normal distributions $\sigma_{i}^{2}=\left\{0,10^{-4},10^{-3},10^{-2}\;\right\}\times \sigma_{g}^{2}.$ This specification implies that the BayesR model assigns a mixture prior of normal distributions for the SNP effects so that every SNP can belong to any of these distributions. Being a generalization of BayesC (Habier et al., 2011), the first distribution is point-mass at zero to account for SNPs with no effect in the phenotype. **V** and **Z** are design matrices of farm effects (*n* × *p*) and standardized genotypes (*n* × *m*), respectively. Finally, **e** is a vector of random residuals with a distribution $e\&sim;N\left(0,E\sigma_{e}^{2}\right),$ where **E** is a diagonal matrix (1/*w_i_*) and *w_i_* is a weight for each animal. Weights were calculated following the formula derived by Garrick et al. (2009) for cows:

[2] $$w_{i}=\frac{r_{i}\left(1-h^{2}\right)}{1+\left(r_{i}-1\right)t-r_{i}h^{2}},$$

where *r_i_* is the number of records for animal *i*, *h*^2^ is the heritability of the trait (single records), and *t* the trait repeatability. Again, we set *t* = 0.60 and *h*^2^ = 0.19 based on estimates for these genetic parameters in India (Table 3 in Al Kalaldeh et al., 2021).

The BayesR model simultaneously provides estimates for the *m* SNP effects (*g*), and the additive genetic $\left(\sigma_{g}^{2}\right),$ farm $\left(\sigma_{f}^{2}\right),$ and residual variances $\left(\sigma_{e}^{2}\right).$ Note that it can also accommodate fixed effects other than the overall mean *m*. The model was fitted using the open-source software GCTB (Zeng et al., 2018), available at https://cnsgenomics.com/software/gctb. To compare the performance of the proposed Bayesian model, we also estimated GBLUP and BayesC models in Julia v1.6.2 (Bezanson et al., 2017) using the package JWAS (Cheng et al., 2018). All models were run in a Linux server (Intel Xeon 6130, 2.10 GHz, and 314 GB of RAM).

## RESULTS

### Simulations

**Figure 1:**
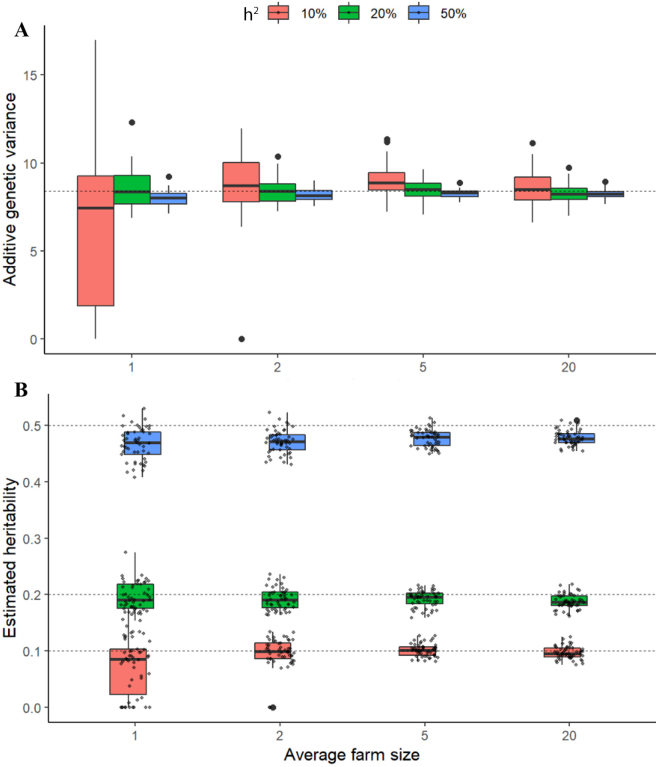
Estimated genetic parameters for simulated phenotypes based on real genotype data. Estimated additive genetic variance ${\hat{\sigma }}_{g}^{2}$ (A) and heritability ${\hat{h}}^{2}$ (B). True values are shown by dashed lines and estimated values as boxplots. Upper and lower edges of boxplots show first and third quartiles, and midline shows the median. Whiskers extend to the largest (or smallest) value no further than 1.5 times the interquartile range. Outliers are represented individually as big dots. Estimated values for all simulations are depicted as small dots in panel B.

The estimated genetic parameters for the simulations based on real genotypes are shown as Figure 1. The true values for these parameters are shown in dashed lines and the estimated values across simulations as boxplots. Estimates for the additive genetic variance $\left({\hat{\sigma }}_{g}^{2}\right)$ are shown in Figure 1A and estimates for the heritability $\left({\hat{h}}^{2}\right)$ in Figure 1B. Estimates for the additive genetic variances are very close to their true values (dashed line) across most scenarios (Figure 1A). For instance, across all simulations the true values for the additive genetic variances are within the first and third quartiles (25th and 75th percentiles) of the distribution of estimated values. However, estimated additive genetic variances have lower accuracy for the scenarios with lower true heritability and smaller farm sizes. For instance, with an average farm size of 1 animal per farm and *h*^2^ = 10%, the values for ${\hat{\sigma }}_{g}^{2}$ are extremely noisy, sometimes including zero. The values for ${\hat{\sigma }}_{g}^{2}\;$ become more accurate with larger farm sizes and true heritabilities. For the scenario with 20 animals per farm on average, ${\hat{\sigma }}_{g}^{2}\;$ is very close to the true value, even for the lowest heritability scenario (*h*^2^ = 10%).

Similarly, the estimated values for the heritabilities are very close to their true values (dashed lines) across all scenarios (Figure 1B). The accuracy of the estimates is lowest with small farm sizes and lower true heritability, with the extreme being the scenario with the lowest heritability and farm size (*h*^2^ = 10%, and 1 animal per farm). The values for ${\hat{h}}^{2}$ become more accurate with increasing average farm size and true heritability, with a greater effect of farm size. Importantly, an average farm size of 2 animals and a true heritability of 20% seems to be sufficient for an accurate estimation of the heritability. This is important because this scenario with very small number of animals per farm is typical in many smallholder systems.

### Validation for the Simulations

**Figure 2:**
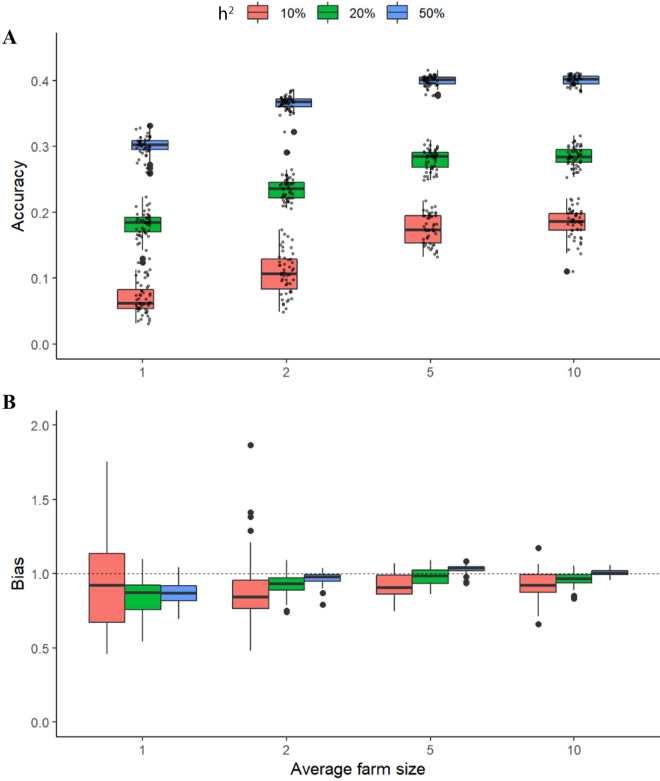
Prediction accuracy (A) and bias (B) from 5-fold cross-validation for genomic prediction for simulated phenotypes based on real genotyped data. Accuracy is defined as the Pearson coefficient (r) and bias as the slope of the regression between the simulated phenotypes and genomic (G)EBV. Dashed line at 1.0 corresponds to unbiased GEBV. Upper and lower edges of boxplots show first and third quartiles, and midline shows the median. Whiskers extend to the largest (or smallest) value no further than 1.5 times the interquartile range. Outliers are represented individually as big dots. Estimated values for all simulations are depicted as small dots in panel A.

Prediction accuracy and bias for the simulations based on real genotypes are shown in Figure 2. The prediction accuracy of the GEBV increases with farm size and true heritability across all scenarios. However, this increase in accuracy is not linear and seems to plateau after an average farm size of 5 animals. For instance, prediction accuracies plateau at approximately 0.18, 0.29, and 0.40 for true heritabilities of *h*^2^ = 10%, 20%, and 50%, respectively. Given the sample size used for this simulation (4,655 cows), these relatively small values for the accuracies are not unexpected. For typical values of smallholder farms in the real data, a heritability of approximately 20% and an average farm size of approximately 2 animals, the expected prediction accuracy is close to 0.22 (Figure 2A).

The prediction bias is also smaller with increasing farm size and heritability, although the trend is less clear when *h*^2^ = 10% (Figure 2B). That is, the higher the heritability and the greater the number of cows in the farm, the closer the predicted values are to the simulated phenotypes. For the extreme scenario with the lowest heritability and farm size, *h*^2^ = 10% and average farm size = 1, the GEBV are deflated on average.

### Real Data Application

The parameters estimated for all genetic models, BayesR, BayesC, and GBLUP, are shown in Table 1. For the baseline model, we estimate very similar additive genetic, farm, and residual variances with all 3 models, which result in heritabilities of 0.16 (0.03) for BayesR, 0.15 (0.02) for BayesC, and 0.16 (0.02) for GBLUP.

**Table 1:** Estimated parameters (SD) for the different genetic models evaluated (BayesR, BayesC, and GBLUP^1^) for monthly test day milk yield records from smallholder crossbred dairy farms in India

Parameter	Baseline (model 1)			+Environment (model 2)		
	BayesR	BayesC	GBLUP	BayesR	BayesC	GBLUP
Additive genetic variance, $\sigma_{g}^{2}$	0.49 (0.08)	0.46 (0.06)	0.49 (0.07)	0.48 (0.10)	0.43 (0.06)	0.51 (0.07)
Residual variance, $\sigma_{e}^{2}$	2.66 (0.14)	2.68 (0.12)	2.64 (0.12)	2.55 (0.15)	2.60 (0.12)	2.52 (0.12)
Farm variance, $\sigma_{f}^{2}$	1.94 (0.07)	1.75 (0.09)	1.74 (0.09)	1.25 (0.07)	1.17 (0.07)	1.15 (0.07)
Heritability, h2=σg2/σg2(σg2+σe2)−(σg2+σe2)	0.16 (0.03)	0.15 (0.02)	0.16 (0.02)	0.16 (0.04)	0.14 (0.02)	0.17 (0.02)
Low production environment				−0.76 (0.05)	−0.75 (0.05)	−0.77 (0.05)
Medium production environment				0.84 (0.07)	0.84 (0.07)	0.84 (0.07)
High production environment				2.01 (0.08)	1.16 (0.08)	1.19 (0.08)

1 GBLUP = genomic BLUP.

For all models, including production environment (model 2) barely changes the estimates for the additive genetic and residual variances, and thus the heritabilities, but greatly reduces the farm variance. For instance, for the BayesR model, it reduces from 1.94 (0.07) to 1.25 (0.07). As expected, the effect of production environment (low, medium, high) in the milk yield phenotype is statistically significant and increases monotonically. This effect is negative for animals in low production environments and positive in animals in medium and high environments, with the latter being the highest. Using the BayesR model, we estimate a heritability of 0.16 (0.04) in a model that also includes production environment (Table 1).

**Figure 3:**
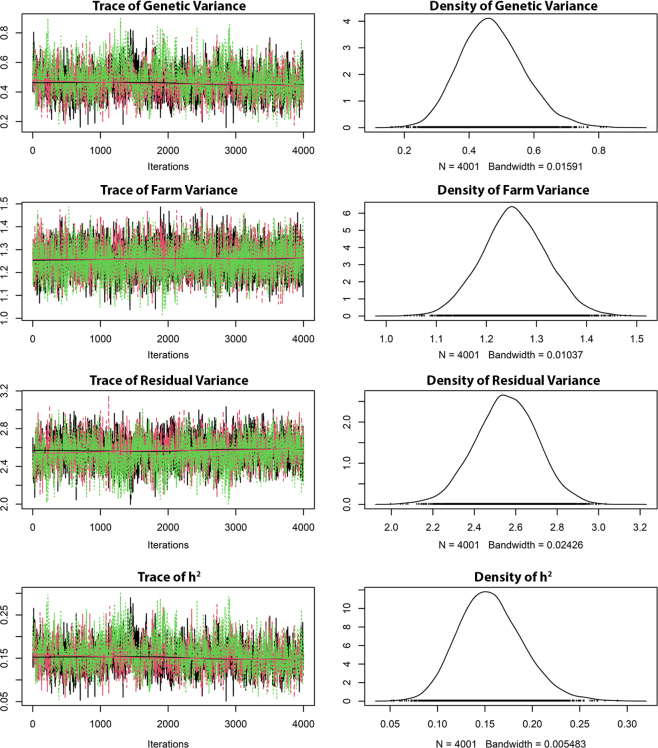
Markov chain Monte Carlo convergence for all chains for the genetic parameters of the BayesR model with farm random effects and production environment (model 2).

The parameters of the BayesR model showed MCMC convergence and good mixing for all chains (Figure 3). The multivariate Gelman-Rubin diagnostic had a value of 1.02. Running times were about 11 h for the BayesR models, 13 h for the BayesC models, and 25 min for the GBLUP models. In terms of peak RAM memory, the BayesR models in GCTB needed 13 GB, whereas the GBLUP and BayesC models used approximately 64 GB.

We also tested breed effects in the genetic models, but they were not significant after accounting for production environment and therefore are not presented here. In what follows, we used the model with production environment (model 2) for QTL mapping and genomic prediction.

**Figure 4:**
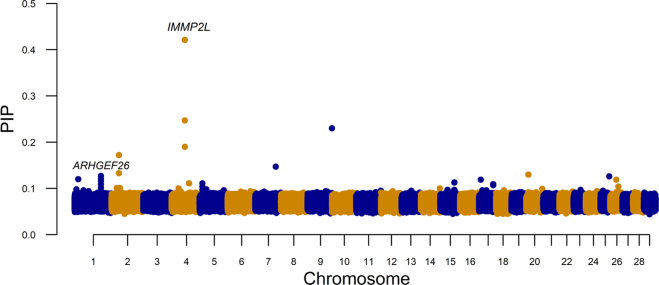
Posterior inclusion probability (PIP) in the BayesR model with farm random effects and production environment (model 2). Marker in chromosome 1 is an intron for *ARHGEF26*, and marker in chromosome 4 is within 100 kb of *IMMP2L*.

The variant with the highest PIP is located in chromosome 4 (rs109218186, minor allele frequency = 0.07), within 100 kb of the mitochondrial inner membrane protease subunit 2 (*IMMP2L*) gene (Figure 4). This gene has previously been associated with milk protein composition (Dadousis et al., 2017) and mastitis resistance (Cai et al., 2018) in dairy cattle. The top variant in chromosome 1 (rs42222474, minor allele frequency = 0.15) is an intron for the rho GTPase activating protein 26 (*ARHGEF26*) gene previously associated with milk cholesterol content (Do et al., 2018). This variant is the only intronic variant among the top 10 examined here.

### Validation for the Real Data Application

The results for the GEBV cross-validation for the real data application using random CDC, including the number of animals in each subset, prediction accuracy, and bias, are presented in Table 2. The number of animals in these random groups varies from 316 to 842, with a mean of 466. On average, for the BayesR model with random farm effects, GEBV accuracy is 0.26 with a bias of 1.13 (0.19). When production environment (model 2) is incorporated, this prediction accuracy reduces to 0.18. Adjusted by heritability, we estimate a GEBV prediction accuracy of 0.45 (0.18/0.180.160.16) for the model with production environment as a fixed effect using this cross-validation across CDC. By comparison, the prediction accuracy of mass selection was 0.55. In addition, prediction bias is the lowest for the model with environment 1.04 (0.26). On average across all CDC, the confidence interval for the bias estimate includes 1, showing evidence that the GEBV are unbiased on average.

**Table 2:** Cross-validation results by cattle development center (CDC) for the BayesR models^1^

CDC group	No. animals	Baseline (model 1)			+Environment (model 2)		
		Accuracy (r)	Bias	SE	Accuracy (r)	Bias	SE
1	399	0.14	0.51	0.18	0.07	0.36	0.24
2	427	0.18	0.58	0.15	0.10	0.46	0.21
3	412	0.23	1.08	0.23	0.21	1.64	0.37
4	465	0.30	1.31	0.19	0.19	0.98	0.23
5	566	0.22	0.84	0.16	0.12	0.62	0.21
6	533	0.37	1.74	0.19	0.34	1.79	0.21
7	316	0.33	1.44	0.24	0.22	1.28	0.32
8	842	0.37	2.13	0.19	0.30	2.10	0.23
9	324	0.29	1.08	0.20	0.16	0.77	0.26
10	371	0.17	0.60	0.18	0.08	0.45	0.29
Average	466	0.26	1.13	0.19	0.18	1.04	0.26

1 SE = standard error for the bias.

## DISCUSSION

We demonstrated by simulations that it is possible to separate genetic effects in smallholder farms using a flexible Bayesian model that simultaneously accounts for nongenetic random effects for farms and varying SNP variance. The model also provides a good way to produce interim GEBV for candidates or newly genotyped animals directly using estimated SNP effects, which provide a prediction equation. The proposed BayesR model is implemented in GCTB, a very efficient, freely available software.

The application to monthly TD milk records of 4,655 crossbred cows in India, with 106,109 records, 689,750 autosomal SNPs, and an average farm size of 1.7 animals per farm, yields an estimated heritability of 0.16 (0.04) in a model that also includes production environment. Using cross-validation, we estimated a GEBV prediction accuracy of 0.45 and a bias of 1.04 (0.26). These estimates are consistent with those obtained using a GBLUP approach, 0.42 in Al Kalaldeh et al. (2021), as well as the accuracy of mass selection (0.55). Genetic gains using genomics can therefore be substantial if GEBV are used to shorten generation intervals. For instance, if the generation interval reduces by half when using GEBV and the other parameters are constant in the breeder's equation, the genetic gain will increase by 64%. When comparing our estimates with those found in other smallholder systems, we found that these accuracies are very similar to those obtained for milk yield in Kenya, 0.32 to 0.41 (Brown et al., 2016), and Tanzania, 0.53 to 0.59 (Mrode et al., 2021). These studies in Africa used smaller reference populations and milk yield records but also obtained smaller estimated heritabilities for this trait.

Our study has some limitations. First, we did not fit breed of origin models (Vandenplas et al., 2016), because they only show small improvements in accuracy of estimates compared with models that ignore breed of origin ( Sevillano et al., 2017; VanRaden et al., 2020; Eiríksson et al., 2022). In smallholder settings, crossbreeding is more complex than in intensive or pasture-based production systems. For example, under Indian smallholder production, the crossbreeding program involves crossing of large numbers of indigenous cattle population, which are not categorized into specific breeds, with Indian dairy breeds (Sahiwal, Gir, Red Sindhi, Tharparkar, and Deoni) as well as with exotic dairy breeds, predominantly Holstein/Friesian and Jersey, resulting in different levels of admixture in the crossbred animals (Strucken et al., 2021).

Crossbreeding also increases the need for bigger reference populations, as the inclusion of genetically divergent breeds can reduce prediction accuracy in genomic evaluations (Makgahlela et al., 2013; Calus et al., 2014) likely due to differences in causal variants and linkage disequilibrium patterns between markers and QTL across breeds. Furthermore, the results' similarity between the different approaches taken here, GBLUP, BayesC, and BayesR, also suggests that a much larger training population might be required for an accurate genetic evaluation in the presence of large environmental effects and crossbreeding and to exploit all the information provided by the high-density genotyping. One related limitation, and the reason why a forward-validation was not carried out, is that no new animals were genotyped in 2020. In addition, only 402 animals were genotyped in 2019, but they were from different geographical locations (farms, CDC, and states). We thus opted for the CDC cross-validation strategy presented here. As sample size grows, in terms of number of animals and records, this limitation will be alleviated.

Another potential limitation of BayesR, and other genetic models that rely on animals with both genotypic and phenotypic information, is that farmers with phenotypic records and (historical) pedigree information will have no GEBV. The problem is complex because single-step GBLUP (Misztal et al., 2009) relies on accurate pedigree recording for several generations, which is not the case for smallholder systems and further parameter calibration to make both pedigree and genomic relationships compatible. Additionally, SSGBLUP models have provided similar results to GBLUP in other smallholder contexts (Mrode et al., 2021). Therefore, no perfect solution exists, and we believe in continuing the research efforts to develop genetic models that, although imperfect, could provide the best performance for a given context.

Typically, Bayesian models also require longer running times than the conventional GBLUP. For the Indian data set at hand (4,655 animals and 689,750 SNPs), although the proposed BayesR model was 1.2 times faster than BayesC (11 vs. 13 h), it was also 26.4 times slower than the conventional GBLUP (11 h vs. 25 min). In terms of computer memory, the proposed BayesR model only needed about 20% of the peak RAM memory (13 GB) required by the BayesC and GBLUP models (64 GB). However, this lower RAM consumption might reflect an efficient software implementation of BayesR in GCTB, compared with a general-purpose package such as JWAS in Julia, rather than intrinsic differences between these genetic models.

Finally, we might not have captured environmental and management effects adequately for milk yield, overcorrecting in the model by using both farm and production environment and reducing the estimated heritability. However, we have identified 2 candidate genes, *IMMP2L* and *ARHGEF2*, and observed a prediction accuracy of 0.45 for this trait. These results are also consistent with those from a previous study using a GBLUP approach for the same data.

Taking these results together, BayesR provided similar genetic parameters and prediction accuracies but was computationally slower than GBLUP for the given Indian dairy smallholder data. Despite smaller accuracy of prediction than mass selection, both genetic models could provide greater genetic gain due to shorter generation intervals. In research settings, where running time and computational resources are not necessarily binding constraints, both models are good alternatives. However, in routine genetic evaluations, time and computational resources could be important factors to consider when assessing the adoption of a genetic model. Currently, we are actively exploring alternative ways of incorporating environmental and breed effects in the genetic models for these smallholder dairy systems.

## CONCLUSIONS

We demonstrated by simulations based on real genotypes that, using the proposed Bayesian model, it is possible to separate genetic effects in smallholder farms, even when farm sizes are small (2 cows on average). For the case study of milk yield from Indian smallholder farms, all 3 genetic models (BayesR, BayesC, and GBLUP) provided similar genetic parameters. The heritability and GEBV accuracies for milk yield are much lower than those usually obtained in intensive or pasture-based systems. Further increases in the number of phenotype and genotyped animals in farm with at least 2 animals (preferably 3–5, to allow for dropout of cows) are needed to improve the accuracy of estimated genetic and farm effects in these smallholder dairy farms.
